# Phenanthrene-enriched extract from *Eulophia macrobulbon* using subcritical dimethyl ether for phosphodiesterase-5A1 inhibition

**DOI:** 10.1038/s41598-022-08553-x

**Published:** 2022-04-09

**Authors:** Jukkarin Srivilai, Panatpong Boonnoun, Tongchai Saesong, Chitaporn Pingyod, Nattiya Chaichamnong, Jinutda Engsuwan, Prapapan Temkitthawon, C. Norman Scholfield, Nitra Nuengchamnong, Nantaka Khorana, Kornkanok Ingkaninan

**Affiliations:** 1 Research and Innovation Center in Cosmetic Sciences and Natural Products, Department of Cosmetic Sciences, School of Pharmaceutical Sciences, University of Phayao, Phayao, 56000 Thailand; 2 Chemical Engineering Program, Department of Industrial Engineering, Faculty of Engineering, Naresuan University, Phitsanulok, 65000 Thailand; 3 Department of Pharmaceutical Chemistry and Pharmacognosy, Center of Excellence in Cannabis Research, Faculty of Pharmaceutical Sciences, Center of Excellence for Innovation in Chemistry, Naresuan University, Phitsanulok, 65000 Thailand; 4 Division of Applied Thai Traditional Medicine, Faculty of Public Health, Naresuan University, Phitsanulok, 65000 Thailand; 5 Akkhraratchakumari Veterinary College, Walailak University, Thasala District, Nakhon Si Thammarat, 80160 Thailand; 6 Science Laboratory Centre, Faculty of Science, Naresuan University, Phitsanulok, 65000 Thailand; 7 Research and Innovation Center in Cosmetic Sciences and Natural Products, Department of Pharmaceutical Sciences, School of Pharmaceutical Sciences, University of Phayao, Phayao, 56000 Thailand

**Keywords:** Biotechnology, Chemical biology, Drug discovery, Biomarkers, Materials science

## Abstract

*Eulophia macrobulbon* (E.C.Parish & Rchb.f.) Hook.f. contains a natural PDE5A1 inhibitor, phenanthrene, 1-(4'-hydroxybenzyl)-4,8- dimethoxyphenanthrene-2,7-diol (HDP), a potential agent for the treatment of erectile dysfunction. The aim of this study was to improve the extraction efficiency of HDP from *E. macrobulbon* by using a more environmentally friendly extraction method, subcritical liquid dimethyl ether extraction (sDME), instead of classical solvent extraction (CSE) and ultrasound-assisted extraction (UAE). The efficiency and quality of the extracts obtained were evaluated using the following criteria: %process yield; solvent amount; extraction time; temperature; %HDP content by LC–MS, bioactivity as inhibition of phosphodiesterase-5A1 (PDE5A1) by radio-enzymatic assay; and chemical profiles by LC-QTOF-MS. sDME provided the highest content of HDP in the extract at 4.47%, much higher than the use of ethanol (0.4–0.5%), ethyl acetate (1.2–1.7%), or dichloromethane (0.7–1.4%). The process yield for sDME (1.5–2.7%) was similar to or lower than the other solvents (0.9–17%), but as long as the process yield is not prohibitively low, the concentration is a more important measure for clinical use. The optimal conditions for sDME extraction were: Extraction time, 40 min; 200% water as co-solvent; sample-to-solvent ratio of 1:8; temperature, 35 °C. Phenanthrene aglycone and glycoside derivatives were the major constituents of the sDME extracts and lesser amounts of phenolic compounds and sugars. The inhibition of PDE5A1 by sDME (IC_50_ 0.67 ± 0.22 µg/ml) was tenfold more potent than ethanolic extract and other extraction methods, suggesting a high probability of clinical efficacy. Thus, sDME was a more efficient, faster, solvent-saving and environmentally friendly extraction method and more selective for phenanthrene when extracted from *E. macrobulbon.*

## Introduction

Erectile dysfunction (ED) or impotence is the inability to achieve penile erection and seriously impinges on the quality of life of patients and their partners^1,2^. Erection occurs following a cascading reaction triggered by nitric oxide released from neural cells. This, which leads to an increase in 3′,5′‐cyclic guanosine monophosphate (cGMP), a pleotropic cell signaling molecule, and ultimately to relaxation of vascular smooth muscle leading to increased blood flow in penile. The cGMP action is curtailed by a large family of phosphodiesterases (PDEs), of which PDE5A1 predominates in penile erection^3,4^. Inhibition of PDE5A1 results in accumulation of cGMP and sustained penile erection. Sildenafil, commonly sold under the brand name Viagra, is a PDE5A1 inhibitor but causes side effects such as visual disturbances, priapism^5^ nausea, headache, and cutaneous flushing^6^. These side effects are caused by sildenafil effect on other PDEs and the ATP‐binding cassette transporter C5^7^. Thus, there is a need for more selective PDE5A1 inhibitor. Interest in drugs derived from plant-based extraction processes has increased^8^. Several herbal remedies claim efficacy for ED including *Panax ginseng* C.A.Mey*., Lepidium meyenii* Walp., *Ferula hermonis* Boiss.*,* and *Ginkgo biloba* L.,^9–13^. The orchid *Eulophia macrobulbon* (E.C. Parish & Rchb.f.) Hook.f. also displays PDE5A1 inhibition embodied in its phenanthrenes, particularly 1-(4'-hydroxybenzyl)-4,8-dimethoxyphenanthrene-2,7-diol (HDP)^14^. *E. macrobulbon* relaxes human corpus cavernosal muscle *in vitro*^15,16^, relaxes rat pulmonary arteries ex vivo and reduces experimental pulmonary hypertension in rats^17,18^. Traditionally, *E. macrobulbon* is an aphrodisiac. Indeed, it promoted erection in aged male rats at a dose of 15mg/kg for 21 days^15^. In addition, anti-inflammatory and antioxidation effects of *E. macrobulbon* extract have also been reported^19^. Taken together, these studies suggest that extraction of HDP from *E. macrobulbon* is likely to lead to promising clinical applications. All previous studies on HDP/*E. macrobulbon* have suggested moderately low doses or extract concentrations and clinical applications of *E. macrobulbon*. Nevertheless, the extraction process must be simple and selective for the therapeutically active compound(s)/target compound while minimizing the inedible and toxic components to improve efficacy, safety and cost. Many extraction procedures for plant-based compounds are tedious, resource intensive and time consuming which limits the use of natural products^20^. Nowadays, extraction methodology with green and sustainable effect have gained much attention from among researchers.

In addition, there is increasing pressure to limit the use of nonpolar solvents, such as hexane and dichloromethane for the extraction of active consituents of herbal feedstock, thereby reducing the environmental impact^21^. Supercritical fluid CO_2_ extraction has been applied for extraction of several plants^22–24^, but the high operating pressure needed imposes prohibitive energy needs^25,26^. As an alternative, liquid dimethyl ether (DME) has several favorable properties for extracting non-polar/semi-polar compounds^25,26^, (i) easy to liquefy and store in light-weight canisters, (ii) relatively inert including towards ozone and relatively resistant to auto-oxidation, unlike other alkyl ethers^27^, (iii) appears to have low toxicity, (iv) synthesizes from biomass on an industrial scale, (v) absorbs 1.5% water thus avoiding pre-drying of the fresh plant^25,28,29^. Accordingly, it is approved for the food and cosmetic/pharmaceutical industries by the European Food Safety Authority^30^ and has been used for the extraction of some plant materials^26,31,32^. At ambient pressure and temperature, DME is a gas (boiling point −24 °C), the saturated vapor pressure at 20°C is 0.51 MPa, thus readily removed by a depressurized step leaving the final product free of solvent^28^. Thus, liquefied DME offers many advantages over a wide range of commonly used solvents.

The application of subcritical (liquefied) DME for extractions has not been previously applied to *E. macrobulbon* roots. Therefore, this study aims to compare the enrichment of bioactive constituents from *E. macrobulbon* by liquefied DME with classical solvent maceration and with/(without) ultrasound-assisted extraction. The chemical identity of the bioactive contents (HDP content) of the extracts, the inhibition of PDE5A1 activity and the chemical constituent profiles were also characterized.

## Results and discussion

### Extraction of *E. macrobulbon* root

The major bioactive compound in *E. macrobulbon* is a phenanthrene, 1-(4'-hydroxybenzyl)-4,8-dimethoxyphenanthrene-2,7-diol (HDP), and has been reported to be a potent PDE5 inhibitor^14^. HDP is approximately 50-folds more potent than the next strongest PDE5 inhibitor among the compounds isolated from *E. macrobulbon* and was therefore used as the main biomarker for this study. In herbal extraction, the yield of the extract as a percentage of the process yield (Y) from the starting material is commonly used to gauge extraction efficiency. Since, the amount of solvent used (v) and extraction period (t) are important determinants of the extractable amounts, efficiency is defined as Y/v or Y/t. However, process yield is not a measure of the purity or concentration of the resulting extract. For medicinal purposes, it is often important to assess the concentration of the biologically active compound within the final extract. Here, we measured the %HDP in the resulting extract (B). Low %HDP values would necessitate further purification or could cause unpredictable therapeutic efficacy. Thus, the yield of the target bioactive compound depends on B/v and B/t. Furthermore, the extraction recovery of HDP, the extractable amount of HDP from dried plant was calculated and compared in units of mg/kg.

Extraction by classical methods: From our preliminary experiments with non-polar solvents, hexane and DCM with polarity indices of 0.1 and 3.1 respectively. The extract from hexane presented negligible amount of both HDP content and process yield, only DCM showed acceptable bioactive HDP content but poor process yield (~1%) (Table 1). However, DCM is classified as carcinogenic organic solvent. The safer, ‘greener’ solvents, EtOH and EtOAc used for classical solvent assisted extractions were compared^33^ and presented the polarity indices of 5.2 and 4.4 respectively. cEtOH provided 13-18% of the total extract (Y), but the HDP content was very low at ~0.5%. The corresponding Y values for EtOAc were 2.0-2.8% (~1.5% HDP content) and for cDCM 0.9-1.7% (~1% HDP content) (Table 1). Thus, the semi-polar solvents cDCM and cEtOAC more selectively extracted HDP from *E. macrobulbon* than EtOH. The appearance of the EtOH extracts was brown, syrupy while the dark brown to black solid was observed in the extracts of EtOAc and DCM for both classic and ultrasonic methods. Extending the extraction period (24–72 h) tended to slightly increase the process yield for cDCM and cEtOH, but not the consistent bioactive compound yield or HDP content (Table 1). Process efficiency (Y/t) for all three solvents changed little across the three time points. Increasing the amount of all three solvents also increased the process yield (Y) at all time points. The percentage of bioactive compound or HDP content (B) in the crude extract was similarly increased. However, both the Y and B parameters for cEtOH at 72 h appeared unaffected by the increase in solvent. This indicated that the extraction was nearly complete under these two conditions (72 h and a 1:20 sample to solvent ratio). In contrast, 72 h extraction with cDCM and cEtOAc showed further process yields (Y) and %HDP contents. Nevertheless, higher volumes of all three solvents were associated with lower extraction efficiencies Y/v and B/v (Table 1). The extractable HDP amount in mg from one kilogram of dried plants using different solvents was then compared (Table1). The result showed that the overall the extractable HDP amount was greatest for cEtOH (~1000 mg/kg), slightly less for cEtOAc (~400 mg/kg) but miserable for cDCM (~200 mg/kg). However, the extractable mass or crude extract is further used as an ingredient in nutraceutical, cosmetic, pharmaceutical and food industries, so higher bioactive content in crude extract means higher therapeutic efficacy. The extractable mass from EtOH gave very high %process yield but negligible %HDP content in the extract compared to other solvents (Table1). This is attributed to the fact that EtOH was non-specific phytochemical extraction for *E. macrobulbon* while EtOAc and DCM gave better selective HDP extraction.

**Table 1:** Extraction of *E. macrobulbon* root by classical solvent extraction (cDCM, cEtOAc, and cEtOH), ultrasound-assistance (uDCM, uEtOAc, and uEtOH), and subcritical dimethyl ether (sDME).

Sample no.	Extract’n protocol	Extract’n period (t) (h)	Water added %w/w	EtOAc %w/w	Sample/solvent ratio	Extraction temp (°C)	Process yield (Y) **%**w/w	HDP content (B) %w/w	Extractable HDP to dried plant (mg/kg)	Extraction efficiency parameters			
										Y/t	Y/v	B/t	B/v
**1**	cDCM	24	–	–	1:6.5	Ambient	1.15 ± 0.10	0.88 ± 0.01	100.77 ± 0.964	0.05	0.18	0.04	0.14
**2**		24	–	–	1:10	Ambient	1.20 ± 0.23	0.93 ± 0.03	111.45 ± 5.131	0.05	0.12	0.04	0.09
**3**		24	–	–	1:20	Ambient	1.64 ± 0.15	1.39 ± 0.00	227.96 ± 2.296	0.07	0.08	0.06	0.07
**4**		48	–	–	1:6.5	Ambient	0.97 ± 0.13	0.73 ± 0.07	70.60 ± 1.582	0.02	0.15	0.02	0.11
**5**		48	–	–	1:10	Ambient	1.22 ± 0.07	1.04 ± 0.04	127.44 ± 0.520	0.03	0.12	0.02	0.10
**6**		48	–	–	1:20	Ambient	1.59 ± 0.20	1.45 ± 0.11	229.77 ± 4.144	0.03	0.08	0.03	0.07
**7**		72	–	–	1:6.5	Ambient	1.14 ± 0.06	0.86 ± 0.06	97.78 ± 0.306	0.02	0.17	0.01	0.13
**8**		72	–	–	1:10	Ambient	1.11 ± 0.03	0.92 ± 0.00	102.53 ± 0.119	0.02	0.11	0.01	0.09
**9**		72	–	–	1:20	Ambient	1.71 ± 0.22	1.45 ± 0.11	247.49 ± 4.724	0.02	0.09	0.02	0.07
**10**	cEtOAc	24	–	–	1:6.5	Ambient	2.03 ± 0.03	1.19 ± 0.05	240.86 ± 0.074	0.08	0.31	0.05	0.18
**11**		24	–	–	1:10	Ambient	2.38 ± 0.02	1.44 ± 0.02	343.32 ± 0.036	0.10	0.24	0.06	0.14
**12**		24	–	–	1:20	Ambient	2.81 ± 0.27	1.75 ± 0.03	492.74 ± 7.208	0.12	0.14	0.07	0.09
**13**		48	–	–	1:6.5	Ambient	2.47 ± 0.06	1.17 ± 0.07	289.89 ± 0.347	0.05	0.38	0.02	0.18
**14**		48	–	–	1:10	Ambient	2.48 ± 0.20	1.41 ± 0.06	350.02 ± 4.201	0.05	0.25	0.03	0.14
**15**		48	–	–	1:20	Ambient	2.95 ± 0.22	1.68 ± 0.06	494.56 ± 4.780	0.06	0.15	0.03	0.08
**16**		72	–	–	1:6.5	Ambient	2.17 ± 0.11	1.15 ± 0.02	249.14 ± 1.213	0.03	0.33	0.02	0.18
**17**		72	–	–	1:10	Ambient	2.40 ± 0.10	1.27 ± 0.05	305.35 ± 0.961	0.03	0.24	0.02	0.13
**18**		72	–	–	1:20	Ambient	2.84 ± 0.05	1.51 ± 0.06	429.40 ± 0.278	0.04	0.14	0.02	0.08
**19**	cEtOH	24	–	–	1:6.5	Ambient	14.51 ± 1.37	0.47 ± 0.00	674.75 ± 188.6	0.60	2.23	0.02	0.07
**20**		24	–	–	1:10	Ambient	13.93 ± 0.66	0.54 ± 0.01	585.88 ± 44.20	0.58	1.39	0.02	0.05
**21**		24	–	–	1:20	Ambient	17.39 ± 1.43	0.53 ± 0.02	1100.54 ± 255.1	0.72	0.87	0.02	0.03
**22**		48	–	–	1:6.5	Ambient	15.85 ± 0.34	0.50 ± 0.01	798.54 ± 49.42	0.33	2.44	0.01	0.08
**23**		48	–	–	1:10	Ambient	15.96 ± 0.32	0.52 ± 0.03	837.89 ± 10.10	0.33	1.60	0.01	0.05
**24**		48	–	–	1:20	Ambient	17.10 ± 0.69	0.63 ± 0.01	1070.81 ± 125.6	0.36	0.86	0.01	0.03
**25**		72	–	–	1:6.5	Ambient	16.46 ± 1.09	0.57 ± 0.02	933.32 ± 18.30	0.23	2.53	0.01	0.09
**26**		72	–	–	1:10	Ambient	17.56 ± 1.11	0.47 ± 0.00	1057.52 ± 128.50	0.24	1.76	0.01	0.06
**27**		72	–	–	1:20	Ambient	17.60 ± 10.6	0.54 ± 0.01	1093.58 ± 106.21	0.24	0.88	0.01	0.02
**28**	uDCM	40 min	–	–	1:10	40	1.87 ± 0.12	1.24 ± 0.01	284.60 ± 0.84	2.80	0.19	2.29	0.15
**29**	uEtOAc	40 min	–	–	1:10	40	2.80 ± 0.72	0.95 ± 0.08	490.64 ± 8.14	4.20	0.28	2.63	0.18
**30**	uEtOH	40 min	–	–	1:10	40	17.87 ± 0.81	0.53 ± 0.01	831.33 ± 0.83	26.80	1.79	0.70	0.05
**31**	sDME	40 min	200	–	1:8	35	1.55 ± 0.08	4.47 ± 0.21	758.73 ± 1.62	2.33	0.19	**6.71**	**0.56**
**32**	sDME	40 min	40	–	1:8	35	1.88 ± 0.08	3.77 ± 0.20	712.24 ± 1.58	2.82	0.24	**5.65**	**0.47**
**33**	sDME	40 min	40	40	1:8	35	2.74 ± 0.03	3.33 ± 0.40	1026.74 ± 1.24	**4.11**	0.34	**4.99**	**0.42**

Significant values are in bold.

Time (t) is the duration of extraction. Process yield (Y) is %weight of extract in dried root powder. % HDP content in extract (B) was measured by LC–MS. All extractions and analyses were done in triplicate. The data are represented as means, ± SD. Extraction efficiencies were calculated by divided %yield and % HDP content in extract with time and solvent volume (v).

Ultrasonic assisted extraction produced both process yield (Y) and HDP content (B) equivalent to classical extraction using the appropriate solvent and the most extreme protocol conditions, but within only 40 min and a 1:10 sample–to– solvent ratio. Thus, ultrasonics significantly increases the extraction efficiency and the extractable amount of HDP from dried plant (Table 1).

In both conventional and ultrasonic extraction protocols, DCM and EtOAc were the most selective solvents for extraction of the target HDP compound. However, DCM is toxic and reactive in the atmosphere, a property that misaligns with the idea of herbal medicines being natural and healthier than synthetic medicines.

### Optimization of subcritical fluid dimethyl ether extraction

**Figure 1:**
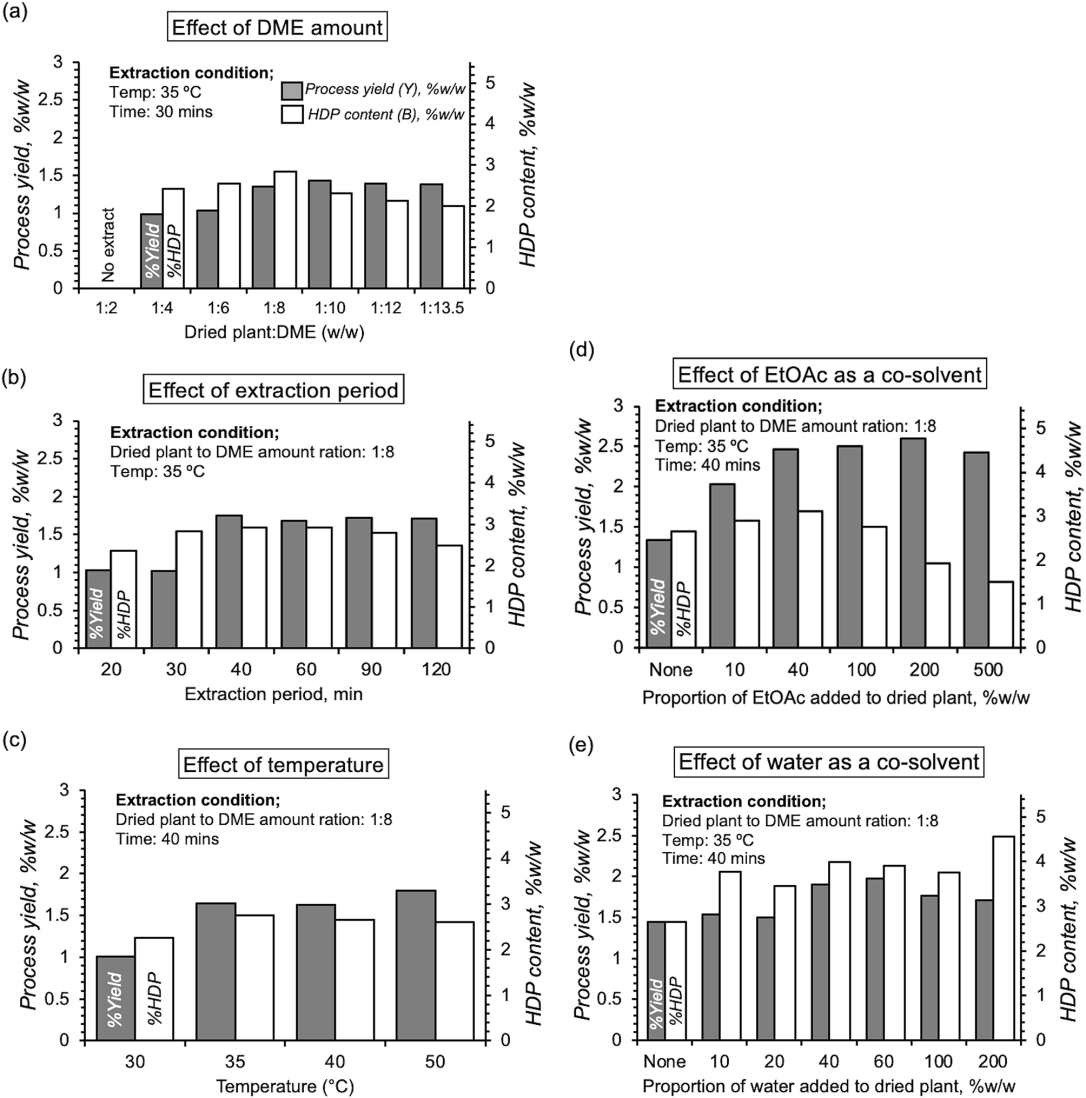
Parameters influencing dimethyl ether (sDME) extraction of *E. Macrobulbon* root powder as total yield (black bars) and its content of the bioactive ingredient, HDP measured LC/MS (open bars). Values deemed optimal for each parameter were used for the next parameter measures **(b–e)** which were **(a)** sDME volume (1:8), **(b)** (40 min extraction period), and **(c)** (35 °C). Each bar is a single determinations.

Subcritical liquid dimethyl ether extraction: Liquid dimethyl ether (DME) is becoming increasingly popular for plant extractions. Here, we explored DME as an alternative solvent to maximize the HDP content and bioactivity of *E. macrobulbon*. We started with a temperature of 35 °C and 30 min as used by others, e.g,^26,31^ and then systematically varied the sample–to–solvent ratio, extraction period, extraction temperature, and adding co–solvents, water, or EtOAc (Fig. 1). The optimum extraction values were selected for each variable and adopted as a fixed value for the next series of determinations.

For each extraction in all protocols using DME, the HDP content was consistently higher than the classical and ultrasonic extraction methods using DCM, EtOAc and EtOH, with increases of ~9-fold, 5-fold and 4-fold respectively. The process yield of the extract with DME was similar to that with EtOAc and DCM, but EtOH extracted a larger bulk (Table1). Fig. 1a showed the effect of sample-to-solvent ratio on the process yield and HDP content. Using DME twice of the dried plant (w/w) (sample-to-solvent ratio 1:2) was not sufficient for the extraction. The sample-to-solvent ratio that provided the optimal content of HDP (~2.8%) was 1:8. Larger solvent volumes or prolonged extraction decreased the apparent content of bioactive HDP as seen elsewhere^34^. This is due to the fact that the overall process yield of the process is increased, while at the same time the risk of undesirable ingredients increases.

The extraction periods were varied from 20 to 120 min and we found that the constant plateau of %yield and %HDP were reached at 40 min (Fig. 1b). The extraction period of 40 min was then selected for further experiments to minimize the time consumption during extraction. The extraction temperatures of 30 to 50 °C were studied (Fig. 1c). It was found that the %yield and %HDP reached the maximum plateau at 35 °C. Therefore, the temperature of 35 °C was selected for further experiments to minimize energy consumption during extraction.

In the classical extraction protocol, EtOAc was an effective solvent for the extraction of HDP and it was classified as a green solvent. So, EtOAc was used as co-solvent in the DME extraction protocol. The result showed that addition of DME with up to ~ 40% EtOAc increased the %HDP in the extract, but further increase in EtOAc content resulted in a decrease in %HDP (Fig. 1d). At 500% EtOAc, the solvent yields an extract with similar properties to one without DME.

Water is commonly used as co-solvent in DME because it is partially miscible in DME solvent and has low cost. Initially, 0.5–10 g of water was added to 5 g of powder, resulting in a sticky mass that increased the process yield of the extract and the HDP yield (Fig. 1e). 10% water was likely absorbed by DME at a pressure and temperature in the extraction chamber^29^ and consumed by hydration of the plant root powder constituents^35^. To our knowledge, the most favorable extraction was observed at 200% water, where most of the water could hydrate or swell the plant matrix, so that DME could easily penetrate to break and extract the plant matrix under pressure^36^. Moreover, the presence of water could lead to higher overall solvent polarity, which ultimately improved the extraction process^37^.

The low extractant concentration in the aqueous phase then provides a steep diffusion or unbinding gradient between the hydrated particles. In our experiments, the mixture of both phases was collected and dehydrated, with the DME forming depository for moderately non-polar compounds. Despite this mechanistic uncertainty, DME with 200% added water was 3–5 folds more efficient for HDP content than the next best extraction protocol, cDCM, cEtOAc or uDCM, uEtOAc (Table 1). In addition, the method is fast and requires a fairly economical amount of solvent. Interestingly, the extraction efficiency parameters with B/t and B/v of sDME were about threefold higher than the best classical and ultrasonic assisted protocol (Table 1). The extractable HDP amount or recovery from the dried plant using sDME reached a peak value of ~ 1000 mg/kg which was equivalent to that of cEtOH (~ 1000 mg/kg) and uEtOH (~ 1000 mg/kg). This indicates that sDME could acheive the maximum extraction of HDP from the dried plant. Moreover, the crude extract from sDME exhibited the highest HDP content among the classical and ultrasonic assisted extractions using EtOH, DCM and EtOAc.

### Chemical profiles by LC–MS/MS

**Figure 2:**
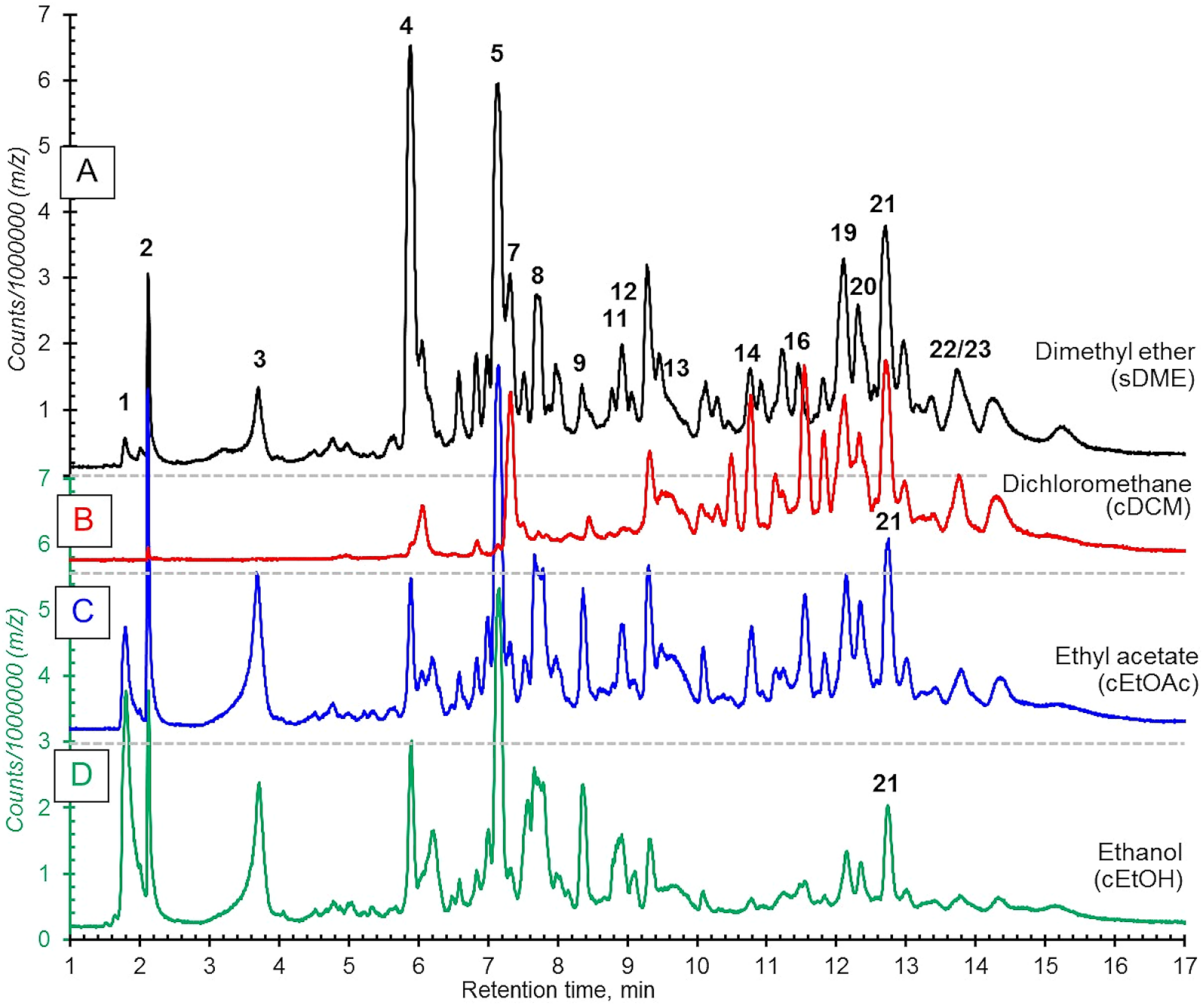
Total ion count LC–MS chromatograms (TIC) from of sample extracts of *E. macrobulbon* with 50 µg/ml. All chromatograms have the same y-scales but only A and D scales shown. The numbered peaks correspond with compounds identified in Table 2. Extraction protocols were: **(A)** 10 g water added 5 g powdered *E. Macrobulbon* root and extracted with 40 g DME (method of sample no. 31, Table 1); **(B)** cDCM, method of sample no. 3 (Table 1); **(C)** cEtOAc, method of sample no. 12 (Table 1), **(D)** cEtOH, method of sample no. 12 (Table 1).

**Figure 3:**
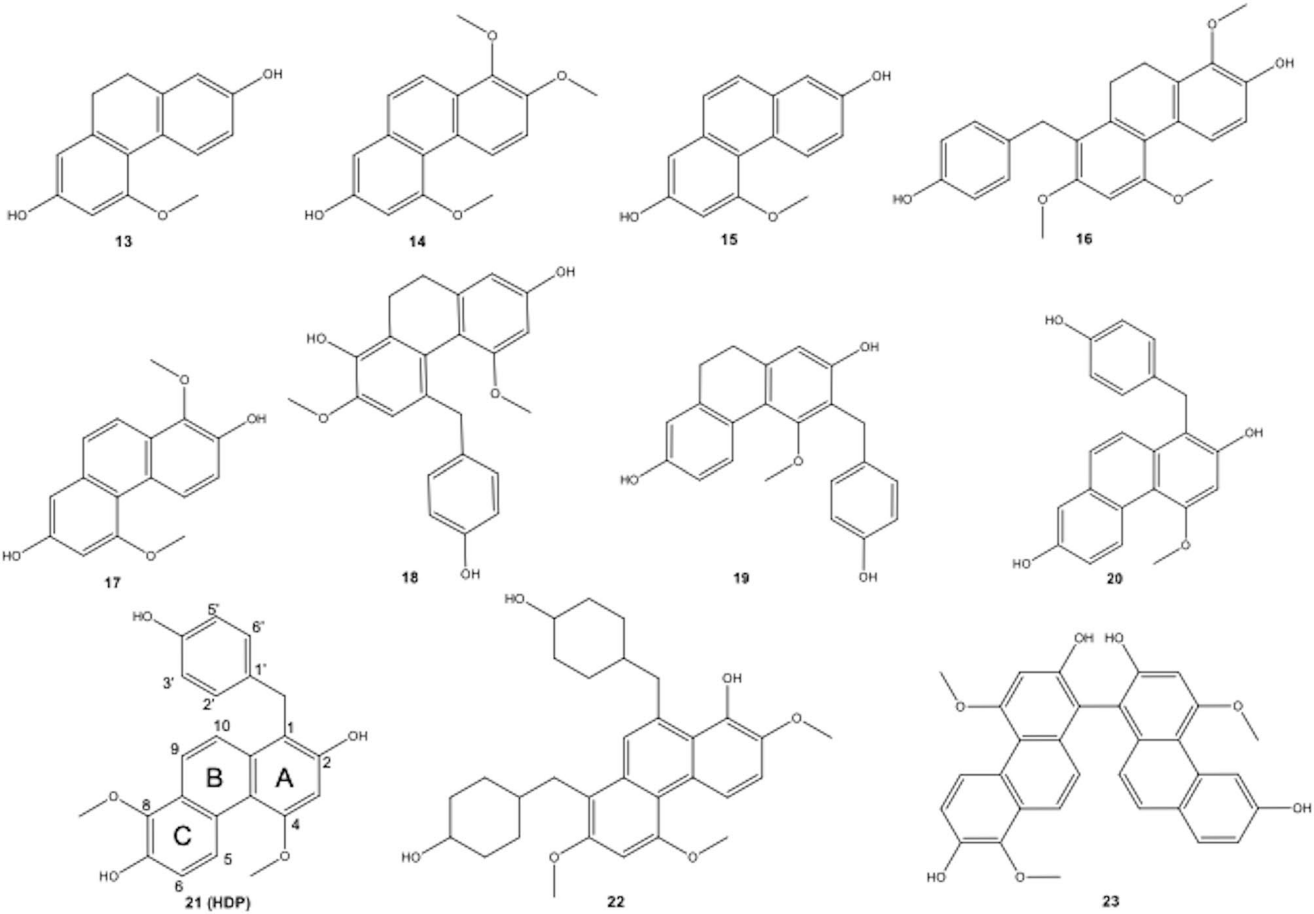
Identifiable compounds of aglycone phrenanthrene structure from *E. Macrobulbon* root extract using LC-QTOF-MS.

The chromatograms of total ion count (TIC) of LC–MS/MS are shown in Fig. 2. Extraction of saccharides (retention time, 1–2 min) was prominent for the more polar solvents (EtOH and EtOAc), whereas DCM extracted only compounds that eluted after ~ 6 min (Fig. 2B). In contrast, EtOH extracted material that eluted mostly before 10 min. For DME extraction, 23 compounds were identified. The compounds of potential pharmacological interest were polyphenols and glycosides (eluted at 3.0–7.5 min) and of current interest, phenanthrenes as glycosides (7.0–9.1 min) and less polar phenanthrene aglycones (9.5–14.5 min) (Table 2). The phenanthrene derivatives were found in the same range with the identifiable peak area in percentage for all extracts, cEtOH, cEtOAc, cDCM and sDME were 62.71, 68.26, 64.76 and 62.68%, respectively. The more polar phenanthrene glycosides were predominantly existed in cEtOH, cEtOAc and sDME with 48.62, 33.55 and 23.77%, respectively. The major phenanthrene glycoside compounds in those extracts were compounds **5**, **8** and **9,** which possess core aglycone mass of 284 [M]^+^, which is the same mass as the aglycone of compound **14**. Only 1.35% of phenanthrene glycosides were found in DCM. This was due to the polarity indices of the solvent for extraction. All mass fragmentations of the identifiable compounds are in Supplementary Data (Table S1). Most phenanthrene aglycones (compound **13**–**21**, see in Fig. 3) were predominantly found in DCM at 63.41%, while phenanthrene aglycones were measured in EtOAc and DME at 34.71 and 38.9%, respectively. Compounds **19** and **21** were reported to have an inhibitory effect on PDE5A1, and compound **21** was identified as HDP, the target PDE5 inhibitor, in the present study^14^. In addition, the toxicity of compounds **13**, **14**, **15** and **21** on human cancerous cell lines was reported, with compound **15** showing the highest toxicity to the human colorectal adenocarcinoma cell line (CaCo-2)^19^. Worth noting, a peak of the natural PDE5A1 inhibitor, HDP (compound **21**) was predominant in sDME with 13.19% of the total identifiable peaks, EtOAC was almost as high with 7.80%. Some phenolic compounds such as 4-hydroxybenzaldehyde and methyl arbutin were found in the extracts (Table 2).

**Table 2:** Identified chemical constituents of *E. macrobulbon* extracts, cEtOH (No. 21), cEtOAc (No.12), cDCM (No. 3), and sDME (No. 31).

Cpd No			Rt (min)		Compound	Ionized mass (*m/z*)	Mass (MW)	Ref	Identifiable peak area, %extract			
									cEtOH	cEtOAc	cDCM	sDME
**1**	Polar comps		1.75		Hexoses	179.0627 [M-H]^−^	180.0700	Lib	0.30	1.33	0.05	0.71
**2**			1.92		Sucrose	387.1266 [M-HCOO]^−^	388.1339	Lib	1.12	0.38	0.01	0.01
**3**			3.69		Methyl arbutin	331.1142 [M-HCOO]^−^	286.1053	Lib	15.37	10.02	0.19	5.79
**4**			5.88		N-Nitroso-3-hydroxypyrolidine	175.0677 [M-H]^−^	176.0750	Lib	13.34	3.73	1.49	12.57
**5**	Glycosides of phenanthrene		7.15		2-ethyl-6-((4,7,8-trimethoxyphenanthren-2-yl)oxy)tetrahydro-2H-pyran-3,4,5-triol	443.1695 [M-H]^−^	444.1768	–	29.35	21.59	0.88	16.99
**6**			7.17		2-((6-ethyl-5-hydroxy-4-((4-hydroxybenzyl)oxy)-2-((4,7,8-trimethoxyphenanthren-2-yl)oxy)tetrahydro-2H-pyran-3-yl)oxy)-6-(hydroxymethyl)tetrahydro-2H-pyran-3,4,5-triol	909.3255 [M + Cl]^−^	874.3348	–	1.49	1.23	0.01	1.10
**7**			7.32		4-Hydroxybenzaldehyde	121.0345 [M-H]^−^	122.0418	–	3.61	6.46	18.91	10.86
**8**			7.69		2-((6-ethyl-5-hydroxy-4-((4-hydroxybenzyl)oxy)-2-((4,7,8-trimethoxyphenanthren-2-yl)oxy)tetrahydro-2H-pyran-3-yl)oxy)-6-(hydroxymethyl)tetrahydro-2H-pyran-3,4,5-triol	711.2709 [M-H]^−^	712.2782	–	9.72	6.76	0.39	3.28
**9**			8.35		2-((6-ethyl-4-((4-hydroxybenzyl)oxy)-5-methoxy-2-((4,7,8-trimethoxyphenanthren-2-yl)oxy)tetrahydro-2H-pyran-3-yl)oxy)-6-(hydroxymethyl)tetrahydro-2H-pyran-3,4,5-triol	761.2647 [M + Cl]^−^	726.2952	–	3.53	2.45	0.04	0.99
**10**			8.80		2-ethyl-6-((2-ethyl-6-((2-ethyl-4-((4-hydroxybenzyl)oxy)-5-((3,4,5-trihydroxy-6-(hydroxymethyl)tetrahydro-2H-pyran-2-yl)oxy)-6-((4,7,8-trimethoxyphenanthren-2-yl)oxy)tetrahydro-2H-pyran-3-yl)oxy)-5-hydroxy-4-((4-hydroxybenzyl)oxy)tetrahydro-2H-pyran-3-yl)oxy)tetrahydro-2H-pyran-3,4,5-triol	1173.4110 [M + Cl]^−^	1138.4621	–	1.11	0.12	0.00	0.02
**11**			8.91		2-((6-ethyl-5-((3-hydroxybenzyl)oxy)-4-((4-hydroxybenzyl)oxy)-2-((4,7,8-trimethoxyphenanthren-2-yl)oxy)tetrahydro-2H-pyran-3-yl)oxy)-6-(hydroxymethyl)tetrahydro-2H-pyran-3,4,5-triol	853.2932 [M + Cl]^−^	818.3234	–	1.32	0.94	0.01	0.81
**12**			9.08		2-((6-ethyl-5-((6-ethyl-3,4-dihydroxy-5-methyltetrahydro-2H-pyran-2-yl)oxy)-4-((4-hydroxybenzyl)oxy)-2-((4,7,8-trimethoxyphenanthren-2-yl)oxy)tetrahydro-2H-pyran-3-yl)oxy)-6-(hydroxymethyl)tetrahydro-2H-pyran-3,4,5-triol	869.3327 [M-H]^−^	870.3400	–	2.10	0.46	0.02	0.58
**13**	Phenanthrene aglycone		9.56		4-methoxy-9,10-dihydro-2,7-phenanthrenediol	241.0881 [M-H]^−^	242.0943	(20)	3.66	3.20	18.25	4.27
**14**			10.73		4,7,8-trimethoxyphenanthren-2-ol	283.0709 [M-H]^−^	284.1049	–	0.49	5.51	9.94	4.44
**15**			11.11		4-methoxy-2,7-phenanthrenediol	239.0719 [M-H]^−^	240.0786	(20)	0.08	1.55	1.91	0.24
**16**			11.46		8-(4-hydroxybenzyl)-1,5,7-trimethoxy-9,10-dihydrophenanthren-2-ol	427.1085 [M-H]^−^	392.1387	–	0.28	0.33	0.14	0.71
**17**			11.53		1,5-dimethoxy-2,7-phenanthrenediol	269.0832 [M + Cl]^−^	270.0892	(20)	0.62	7.61	12.72	3.13
**18**			12.04		(E)-6-((4-hydroxycyclohexa-2,4-dien-1-ylidene)methyl)-1,5-dimethoxy-9,10-dihydrophenanthrene-2,7-diol	377.1402 [M-H]^−^	378.1467	–	0.20	0.57	0.94	2.22
**19**			12.10		1-(4-hydroxybenzyl)-4-methoxy-9,10-dihydrophenanthrene-2,7-diol	347.1399 [M-Cl]^−^	348.1476	(15)	2.46	4.77	8.49	7.11
**20**			12.32		1-(4-hydroxybenzyl)-9-methoxyphenanthrene-2,7-diol	345.1245 [M-H]^−^	346.1205	–	1.49	3.38	2.11	3.60
**21**			12.70		**1-(4-hydroxybenzyl)-4,8-dimethoxy-2,7-phenanthrenediol (HDP)**	375.1361 [M-H]^−^	376.1438	(15,20)	4.81	7.80	8.92	13.19
**22**			14.25		4,4'-((8-hydroxy-2,4,7-trimethoxyphenanthrene-1,9-diyl)bis(methylene))dicyclohexanol	507.1606 [M-H]^−^	508.1679	–	0.95	2.99	4.15	2.28
**23**			14.26		2,5,7-trimethoxy-8,10-bis((4-methoxycyclohexyl)methyl)phenanthren-1-ol	537.1722 [M-H]^−^	538.1795	–	2.58	6.84	10.45	5.10
Total, %									100.00	100.00	100.00	100.00

Lib is Mass Hunter library.

Significance text is given in bold.

### Inhibition of PDE5-1A

The favorable extracts from the different extraction methods were evaluated for their PDE5A1 inhibitory activity by enzymatic and [^3^H]cGMP radioassay, and the result is shown in Table 3. We found that the PDE5A1 inhibition of all extracts was related to the %HDP content in the extract. More potent PDE5A1 inhibitory activity was observed at higher %HDP content (Table 3). This result supports that HDP is a suitable biomarker for the PDE5A1 inhibitory activity of this plant^17^. Moreover, PDE5A1 inhibitory activities of the extracts of the classical method were slightly stronger than those of the ultrasonic-assisted method in all solvents. This is due to the fact that the extracts of the ultrasonic-assisted method contain more undesirable compounds than others. Both extracts of EtOAc and DCM showed more potent PDE5A1 inhibition than EtOH, indicating that DCM and EtOAc with polarity indices of 3.1 and 4.4 could selectively extract PDE5A1 inhibitors than EtOH with polarity indices of 5.5. The extract with DME/200% water showed the most potent PDE5A1 inhibition (Table 3, Fig. S1.) compared to DCM and EtOAc (twofold lower) and EtOH (~ tenfold lower). This confirms that DME (with/without water) is the most selective solvent for PDE5A1 inhibitors. The differences of bioactivity are roughly consistent with the differences in HDP content (Table 1). In addition, other phenanthrenes in *E. macrobulbon* root that are known PDE5A1 inhibitors^14,19^ might play roles.

**Table 3:** Inhibition of PDE5-1A by various extracts and %HDP content (the experiment was done in triplicate).

Extraction method (sample no.)	%Yield ± SD (g/g)	%HDP content (g/g)	IC_50_ (in µg/ml) against PDE5
cDCM (No. 3)	1.64 ± 0.15^#^	1.39 ± 0.001*	1.12 ± 0.09^d^
cEtOAc (No. 12)	2.81 ± 0.27^†^	1.75 ± 0.03**	1.30 ± 0.46^d^
cEtOH (No. 21)	17.39 ± 1.43^‡^	0.53 ± 0.02***	4.03 ± 0.16^c^
uDCM (No. 28)	1.87 ± 0.12^#^	1.24 ± 0.01****	1.24 ± 0.11^d^
uEtOAc (No. 29)	2.80 ± 0.72^†^	0.95 ± 0.08****	1.64 ± 0.17^c^
uEtOH (No. 30)	17.87 ± 0.81^‡^	0.53 ± 0.01***	6.29 ± 0.08^b^
sDME (No. 31)	1.55 ± 0.08^#^	4.47 ± 0.21*****	0.67 ± 0.22^a^

The uppercase symbols stand for significantly different (*p* < 0.05).

Sildenafil was used as positive control and presented IC_50_ at 0.002 ± 0.0008 µg/ml in triplicate.

## Conclusions

This study investigated the potential of phenanthrene enrichment extraction using a more environmentally friendly and safer technique: extraction with liquefied dimethyl ether from *E. marcobulbon*. We found that an optimized sDME protocol with an extraction time of 40 min, addition of 200% water to sDME (%w/w), a sample to solvent ratio of 1:8, and a temperature of 35 °C gave a process yield of 1.55% with an HDP concentration of 4.47% in the resulting extract. The process yield was comparable or in some cases lower than the optimal protocols using cDCM, cEtOAc and cEtOH. However, HDP concentration was dramatically higher using sDME than the best non-DME protocol (cEtOAc gave a maximum HDP concentration of 1.75%), CSE and UAE in all solvents. A high HDP concentration is critical for clinical applications, as higher compound purity is likely to lead to more predictable and effective results. Indeed, we found that the extract obtained with our optimized sDME protocol exhibited approximately tenfold higher efficacy in inhibiting PDE5A1 compared to the uEtOH extract, suggesting a promising clinical application. In addition to a high HDP concentration and promising PDE5A1 inhibition, sDME is a more environmentally friendly and safer solvent than other organic solvents such as DCM, chloroform, petroleum ether, benzene and the others used here. The chemical fingerprint profile of the sDME extract was identified using LC-QTOF /MS and could be classified into 4 main classes: sugars, phenolic compounds, phenanthrene glycosides and phenanthrene aglycones. The main constituent of the extract was phenanthrene derivatives. Thus, the use of sDME is a promising technique for selective enrichment of phenanthrene extract from *E. macrobulbon*.

## Materials and methods

### General materials

Dimethyl ether or DME (Tamiya 420D, commercial grade) was used for extraction and purchased from Siam Tamiya Co., Ltd., Thailand. The cGMP, crude snake venom from *Crotalus atrox*, histone from calf thymus, bovine serum albumin (BSA), ethylene glycol tetra-acetic acid (EGTA), imidazole, Tris ((trishydroxymethyl)aminomethane), magnesium chloride (MgCl_2_), DEAE-Sephadex, phenylmethylsulfonyl fluoride (PMSF) were bought from Sigma‐Aldrich (St Louis, MO, USA) [^3^H]cGMP and scintillation cocktail Ultima gold was purchased from Perkin Elmer (Waltham, MA, USA). Dulbecco's Modified Eagle's Medium (DMEM), fetal bovine serum (FBS), penicillin–streptomycin (Pen‐Strep), and Geniticin (G418) were purchased from Gibco by Life Technologies (Paisley, Scotland). Lipofectamine 2000 (Invitrogen) was purchased from ThermoFischer Scientific (Waltham, MA, USA). A Hipure plasmid Maxiprep kit was bought from ThermoFischer Scientific. Human embryonic kidney (HEK)293 cell lines were purchased ATCC (Virginia,USA). Genistein (A) (purity >98%) was purchased from Apex Biotechnology (Boston, USA). Sildenafil citrate (purity >98%) was purchased from the European Directorate for Quality of Medicines and Health care (EDQM), Council of Europe (Strasbourg, France). ACN, water and MeOH (LC‐MS grade) were purchased from RCI Labscan, (Bangkok, Thailand). Formic acid (AR grade) was obtained from Merck (Darmstadt, Germany). The organic solvents (analytical grade) were purchased from Burdick & Jackson (B&J) (UK). TLC aluminium sheets and silica gel 60 F254 were purchased from Merck (Darmstadt, Germany).

### Plant material

*E. macrobulbon* was collected in Prachinburi province, Thailand. It was identified by Asst. Prof. Dr. Anupan Kongbangkerd, Faculty of Sciences, Naresuan University. The herbarium specimen (No. 002716) is kept in the Biology Department, Faculty of Sciences, Naresuan University, Thailand, which is in compliance with the Convention on Biological Diversity and the Convention on Trade in Endangered Species of Wild Fauna and Flora. The fresh roots were chopped and air-dried at 55 °C for 3 days. The dried plant was ground into fine powder (4 kg) and sieved (150–170 µm) and stored in a desiccator at room temperature until use.

### Isolation of the main bioactive compound from *E. macrobulbon*

The isolation of HDP followed previous reports with some modifications^14^. In brief, dried powders of *E. macrobulbon* (4 kg) were macerated two times with 95% EtOH (28L), then filtered and the solvent was removed under reduced pressure to provide 450 g of crude extract (11.2% yield). The extract (384.4 g) was dissolved in 100% MeOH and partitioned twice with hexane. The hexane part was discarded and the MeOH part was diluted with DI water to give 20% MeOH and partitioned twice with DCM. The DCM portion was dried under reduced pressure to yield 19.9 g of crude extract. The DCM residue was mixed with silica gel and loaded on to a silica gel chromatography column (i.d. 103 × 40 mm). The mobile phase for gradient elution was 100%DCM to 0.5–4% MeOH in DCM. Eighteen fractions were collected (EMD-1-18). The target compound was monitored to reference standard of HDP by TLC using DCM:MeOH (9.5:0.5 %v/v) as the mobile phase (the Rf value was around 0.3). The fraction of EMD-14 was obtained 0.49 g and chosen for further isolation. EMD-14 (0.24 g) was dissolved in methanol and subjected in a Sephadex LH-20 column (i.d. 1.5 × 200 cm) eluting with 100% MeOH to yield 19 fractions. Three fractions (EMDLH14-10 to EMDLH14-12) were pooled and evaporated and recrystallized with MeOH/DCM to give 0.19 g of crystalline bioactive compound (HDP). The spectroscopic data of ^1^H-NMR and MS were in agreement with those reported in the literature^14^. The purity and spectroscopic data of HDP are described in supplementary materials, Figs. S2, S3 and S4. The isolated HDP was used as a reference standard to quantitatively control the quality of the extracts using LC-MS.

### Methods of classical solvent and ultrasound-assisted extraction

Classical solvent extraction: fine powder of *E. macrobulbon* root (10 g) was macerated in different solvents, (i) 95% EtOH, (ii) EtOAc, or DCM. The sample-to- solvent ratio (w/w) was varied from low to high (1:6.25, 1:10 and 1:20), each maceration period was either 24, 48 or 72 h.

Ultrasound-assisted extraction; the fine powder (10 g) was macerated with different organic solvents, EtOH, EtOAc and DCM at a fixed sample-to-solvent ratio of 1:10 at 40 °C for 40 min. The ultrasound frequency was set at low to high intensity (100 kHz to 1 MHz) (Transonic, Themo Fisher Scientific, Göteborg—Sweden). Whenever the extraction process reached the time course, the extraction samples were filtered (Whatman paper 2 µm) and then dried under reduced pressure to provide the crude extract. The extract was then dried over a desiccant for 48 h and weighed.

Subcritical fluid dimethyl ether extraction; the dried powder (5 g) was mixed with the required volume of water or co-solvent and the mixture was placed in cellulose thimble (30 × 100 mm) along with a magnetic bar of 15.9 × 8 mm (length × diameter). The DME extractor was applied for this work and the apparatus was schematically presented in reference of^38^. The thimble was then placed in an extractor (100 ml total volume of stainless-steel with a closed system). Liquefied DME was filled into the extractor at the required solvent to solid weight ratio. The extraction was carried out at a controlled temperature and stirring speed of 500 rpm required time (see below). After extraction, DME and the liquid sample were passed through a stainless steel filter (5 µm pore diameter, Swagelok). The chamber was inverted to a 75 ml Erlenmeyer flask. The remaining liquid sample was then dried over desiccant for 48 h, the amount weighed and the yield determined.

### Optimization of dimethyl ether extraction

The extraction conditions were optimized by comparing;

(i) The amount of solvent as ratio of dried sample to DME solvent (w:w) was varied from 1:2, 1:4, 1:6, 1:8, 1:10, 1:12 and 1:13.5. The optimum ratio was selected for the extraction period.
(ii) The extraction period was varied (20, 30, 40, 60, 90 and 120 min). The minimum time necessary to achieve asymptotic HDP content was chosen.
(iii) The extraction temperatures were set at 30 ± 1, 35 ± 1, 40 ± 1, and 50 ± 1 °C.
(iv) The amount of co-solvents, water of 0%, 10%, 20%, 40%, 60%, 100% and 200%, or EtOAc of 0%, 10%, 40%, 100%, 200% and 500% of the powder weight.

The PDE5A1 inhibitory bioactivity, %HDP content and chemical profile were determined for all extracts. The extraction efficiency was also evaluated using the following parameters;

1. Y; Percentage of process yield (%w/w)
2. B; Percentage of HDP content in the extract (%w/w)
3. t; Extraction period (min)
4. v; Solvent amount
5. Y/v and Y/t
6. B/v and B/t
7. Extractable HDP amount to dried plant (mg/kg)

These parameters were determined for all extraction methods, classical solvent extraction, ultrasound-assisted extraction, and subcritical fluid dimethyl ether extraction.

### Quantitative determination of HDP content in *E. macrobulbon* extracts using LC–MS

A method for the determination of HDP in *E. macrobulbon* samples by LC–MS was developed and validated. An Agilent 1260 infinity Series HPLC coupled to an Agilent-6540 Q-TOF-MS spectrometer was used. The chemical constituents were separated on an EC-C18 (50 × 3 mm, 2.7 cm) column. The mobile phase consisted of 0.1% formic acid in water (A) and 0.1% formic acid in ACN (B). The following gradient system began from 0 to 5 min, 40% and 5–8 min, 20% B with a post run 2 min. The injection volume was 5 µl, flow rate was 0.3 ml/min and the column was maintained at 35 ºC. The optimized MS conditions were: drying gas flow 10 L/min, drying gas temperature 350 °C, nebulizer 30 psig, capillary voltage 3500V, skimmer 65 V, and octapole RFV 750 V. The ESI negative ionization in the Scan and SIM mode was used. The validation data was analyzed by Agilent MassHunter Quantitative Analysis Software Version B.05.02/Build 5.2.365.0. The analytical method was validated and standard curve HDP was established. The stock solution of HDP standard was freshly prepared by dissolving in 100% MeOH to obtain stock concentration of 100 µg/ml. This solution was further diluted with MeOH to make standard concentrations for the creation of calibration curves (0.25, 0.5, 1.0, 2.5, 5.0 and 10.0 µg/ml). Samples were dissolved in 100% MeOH giving solutions of 5 mg/mL, then diluted to 50 µg/ml. All samples and standards were filtered through nylon syringe filters (0.45 μm pore size) before injection. All analyzes were performed in triplicate.

During the analysis, the stability of the LC–MS system was checked by using QC1 (concentration of 1.5 µg/ml) before starting each experimental batch. In addition, QC1 was added for the injections at the beginning, middle and end of the experiment to evaluate the LC–MS system and the stability of HDP throughout the analysis of the sample batch.

### Qualitative analysis of *E. macrobulbon* extracts by LC-ESI-QTOF-MS

Conditions for LC–MS to measure secondary metabolites in *E. macrobulbon* samples were determined using a Zorbax Eclipse Plus C18 (4.6 × 100 mm, 3.5µm) column and gradient elution with 0.1% formic acid in water (A) and 0.1% formic acid in ACN (B). The elution program ran for 0 min, 5%B; 0–6 min, 35%B; 6–10 min, 50%B; and 10–18 min, 20% B with a follow-up time of 2 min (post-run). The flow rate was 0.6 ml/min, the injection volume was 10µl, and the column temperature was maintained at 35 °C.

The MS condition was: negative ESI ionization in scan and SIM mode; drying gas flow 10 L/min at 350 °C; nebulizer 30 psig; capillary voltage 3500 V; skimmer 65 V; octapole RFV 750 V; and fragmentor in negative mode used 250 V. The mass range was set at 100–1200 *m/z* and the collision energy of target MS/MS was operated at 10, 20, and 40V, respectively. Data from LC–MS/MS were acquired using Agilent LC-MS-QTOF MassHunter Data Acquisition Software version B.05.01 and Agilent MassHunter Qualitative Analysis Software B 06.0 for structure elucidation. For the structure elucidation, compounds were compared with previous literature data with ion molecular mass and fragmentation pattern or with MassHunter Metlin Metabolite PCD/PCDL database (Agilent Technologies), from Scifinder (https://scifinder.cas.org), Chemspider (http://www.Chemspider.com) and/or Massbank (http://www.massbank.jp).

Samples from a suitable extraction condition were prepared at 5 mg/ml in 100% MeOH and diluted to 50 µg/ml. They were then filtered through nylon syringe filters with a pore size of 0.45 μm before injection into the LC system.

### Preparation of phosphodiesterase-5 (PDE5-A1)

HEK293 cells were grown in DMEM supplemented with 10% FBS, in 75 mm flasks at 37 °C in a humidified 5%CO_2_. A human PDE5A1 plasmid, a gift from Professor Dr Joseph A. Beavo, University of Washington, Seattle, WA, USA, were sub‐cloned into a pcDNA3 vector containing an ampicillin resistant gene. The human PDE5-A1 plasmid was scaled up and purified using Hipure plasmid Maxiprep kit (Invitrogen‐PureLink). HEK293 cells were transfected with human PDE5A1 plasmid using Lipofectamine-2000 following the company protocol. After 2 days of transfection, PDE5-A1 expression was induced by a selective antibiotic (Geneticin (G418, Gibco), 1 mg/ml) for 7 days. The surviving cells were sub‐cultured in DMEM, supplemented with 10% FBS in 175 mm flasks at 37 °C in a humidified 5% CO_2_ atmosphere, and the cells further cultured until they reached 90–100% confluence. The cells were then harvested using a scraper and lysed by sonication in 1 ml of Tris buffer [50 mM Tris pH 7.5, 2 mM EDTA, 1mM dithiothreitol (DTT) and 1:100 of 100 mM PMSF]. The homogenate was centrifuged at 4 °C for 20 min and the supernatant was used as a source of PDE5A1. A PDE5 inhibitor, sildenafil, was used to confirm the presence of PDE5A1 enzymatic activity.

### Measurement of PDE5-A1 enzyme activity

To assess PDE5A1 inhibition, a reaction mixture comprising 20 µl of reagent A (100 mM TrisHCl (pH 7.5), 100 mM imidazole, 15 mM MgCl_2_, 1.0 mg/ml BSA and 2.5 mg/ml snake venom), 20 µl of 10 mM EGTA, 20 µl of PDE5A1 solution, and either 20 µl of test sample or solvent (5% DMSO) only as a control. The reaction was started by adding substrate 20 µl of 5 µM [^3^H]cGMP (~50,000 cpm) and performed at 30 ºC for 40 min. Then, 100 µl of 50% DEAE resin was added to the reaction. After shaking for 10 min, the resin was allowed to settle (20 min), the supernatant was treated with a second cycle of 50% DEAE resin. This supernatant (100 µl) was shaken with 200 µL of MicroScint-20 and tritium counted on a TopCount NXT scintillation counter (PerkinElmer, USA) for 2 h. The PDE5A1-hydrolyzed <25% of the substrate. Each was performed in duplicate in 96-well plates^27,28^.

In preliminary screening, samples of plant extract and pure compound were tested at final conc of 50 µg/ml and 10 µM respectively. All samples were dissolved in DMSO and diluted with water. DMSO was limited to 1% in the final assay medium. When PDE5A1 inhibition was >80%, samples were further diluted and re-analyzed. IC_50_s were calculated using Prism software (Graph Pad Inc., San Diego, CA). Sildenafil was used as the positive control.

### Data analysis

The %PDE5A1 inhibition was calculated and plotted against log10[sample] and thereafter, half maximum inhibitory concentrations (IC_50_) were interpolated by Graph-Pad Prism v. 8 (San Diego, USA). Data were processed by analysis of variance (ANOVA) or Tukey’s multiple comparison tests. Results were considered significant where *P*<0.05. Means and SDs were all calculated from at least three determinations of each sample.

### Ethics statement

The research did not include human subjects or animal experiments.

## Supplementary Information

Supplementary Information.

## Abbreviations

ACN: Acetonitrile
cGMP: 3′,5′‐Cyclic guanosine monophosphate
CSE: Classical solvent maceration
DCM: Dichloromethane
DEAE: Diethylaminoethyl cellulose
DME: Dimethyl ether
sDME: Subcritical dimethyl ether
DMSO: Dimethyl sulfoxide
ED: Erectile dysfunction
EtOAc: Ethyl acetate
EtOH: Ethanol
HDP: 1-(4'-Hydroxybenzyl)-4,8-dimethoxyphenanthrene-2,7-diol
MeOH: Methanol
PDE5A1: Phosphodiesterase-5A1
UAE: Ultrasound-assisted extraction
