# Supplementary material for: Phenanthrene-enriched extract from Eulophia macrobulbon using subcritical dimethyl ether for phosphodiesterase-5A1 inhibition
Source: Sci Rep. 2022 Apr 9;12:5992. doi: 10.1038/s41598-022-08553-x (PMC8994770; doi:10.1038/s41598-022-08553-x)
Supplement: Supplementary file 1 — Supplementary Information. [file 41598_2022_8553_MOESM1_ESM.docx]

**Phenanthrene-enriched extract from *Eulophia macrobulbon* using subcritical dimethyl ether for phosphodiesterase-5A1 inhibition**

**Supplementary data set**

**1:** Figure S1. PDE5-1A inhibition against concentration (ug/ml) of extracts using different solvents: dimethyl ether (DME), dimethyl chloride (DCM, 1:10 and 1:20), ethyl acetate (EtOAc, 1:10 and 1:20), and ethanol (EtOH, 1:10 and 1:20). Each point are means of single determines from three extracts with each protocol. Bars are ±SEMs (hidden if smaller than symbol).

**
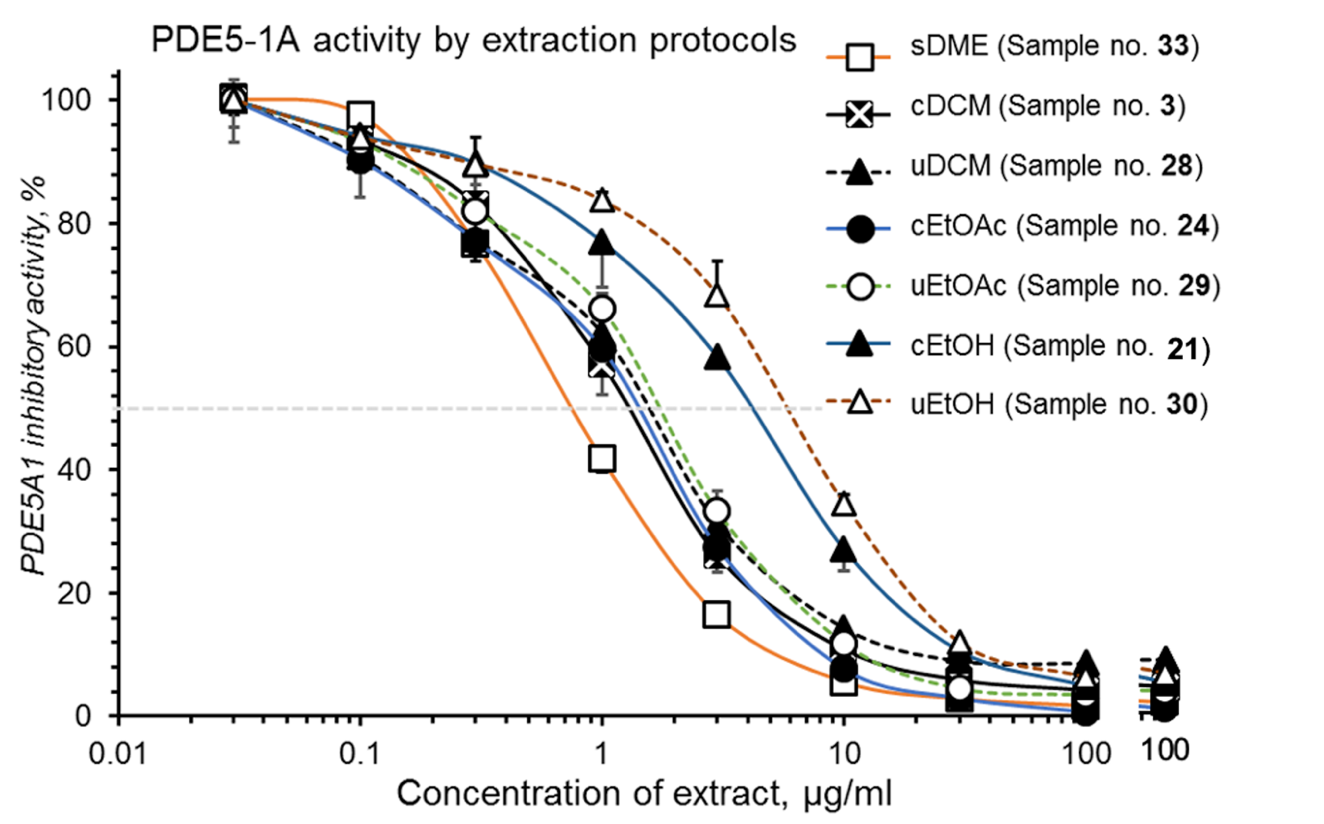
**

**2:** Figure S2. HPLC profile of isolated HDP from E. macrobulbon under UV light at 254 nm (purity > 95%) The separation was carried out on a C18‒HQ (4.6x250 mm, 10 μm) with a guard column (4.6×10 mm, 10 μm). The elution program was achieved with a gradient system of 0.1% formic acid in water (A) and in acetonitrile (B); starting at 0 min, 30% B; 35 min, 45% B; 36 min, 100% B; and 36‒40 min, 100% B for washing. The injection volume was 20 µl with a flow rate at 1 ml/min and 254 nm for detection.


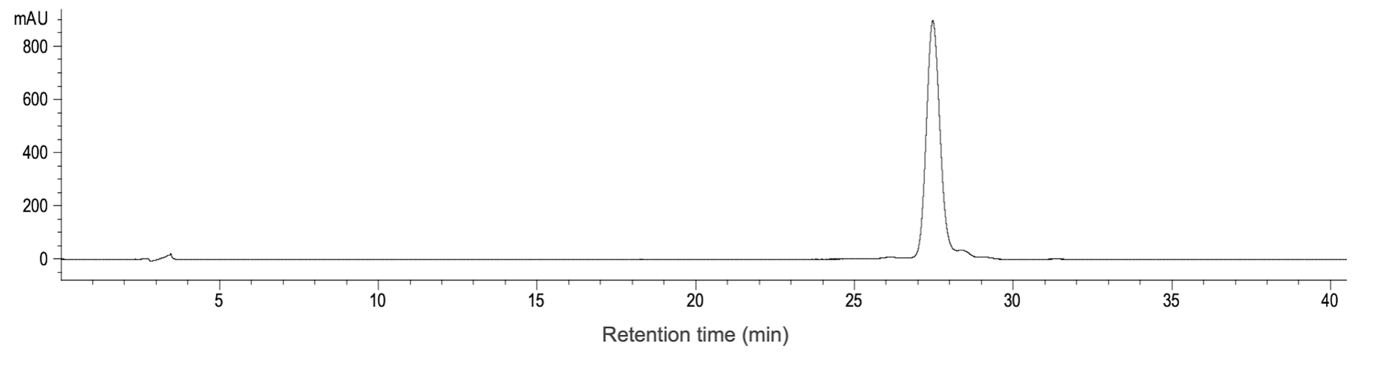


**3:** Figure S3. LC–MS profile of isolated compound 1 in negative ion mode (purity > 95%) The chromatographic separation of isolated compound 1 was carried out on a Waters BEH C18 50×2.1 mm, 1.7 μm column. The mobile phase contained 0.1% formic acid in water (A) and in acetonitrile (B) with flow rate 600 μl/min. The linear gradient elution was begun from 5−100% B for 7 min, followed by an isocratic system at 100% B for 1 min. Injection volume was 1 μl. Mass spectrometry analysis was conducted in negative ion mode.


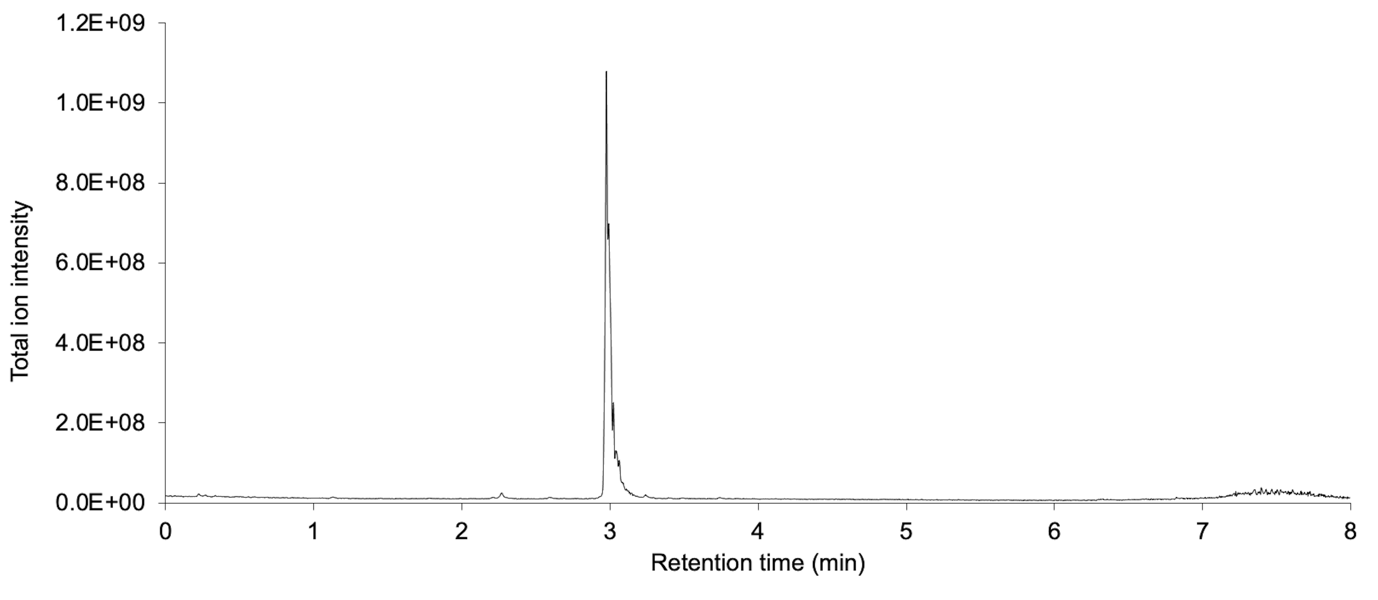


**4:** Figure S4. ^1^H-NMR spectrum of HDP, (Methanol-d_4_, 400 MHz) Bruker AV400 (USA), 1‒(4'‒hydroxybenzyl)‒4,8‒dimethoxyphenanthrene‒2,7‒diol (HDP); ^1^H NMR (600 MHz, MeOD) *δ* 9.22 (d, *J* = 9.4 Hz, 1H, H‒5), 7.91 (d, *J* = 9.5 Hz, 1H, H‒9), 7.82 (d, *J* = 9.5 Hz, 1H, H‒10), 7.14 (d, *J* = 9.4 Hz, 1H, H‒6), 7.02 (d, *J* = 8.5 Hz, 2H, H‒2',6'), 6.90 (s, 1H, H‒3), 6.64 (d, *J* = 8.6 Hz, 2H, H‒3',5'), 4.33 (s, 2H, H‒7'), 4.09 (s, 3H, 4‒OMe), 3.91 (s, 3H, 8‒OMe).

**
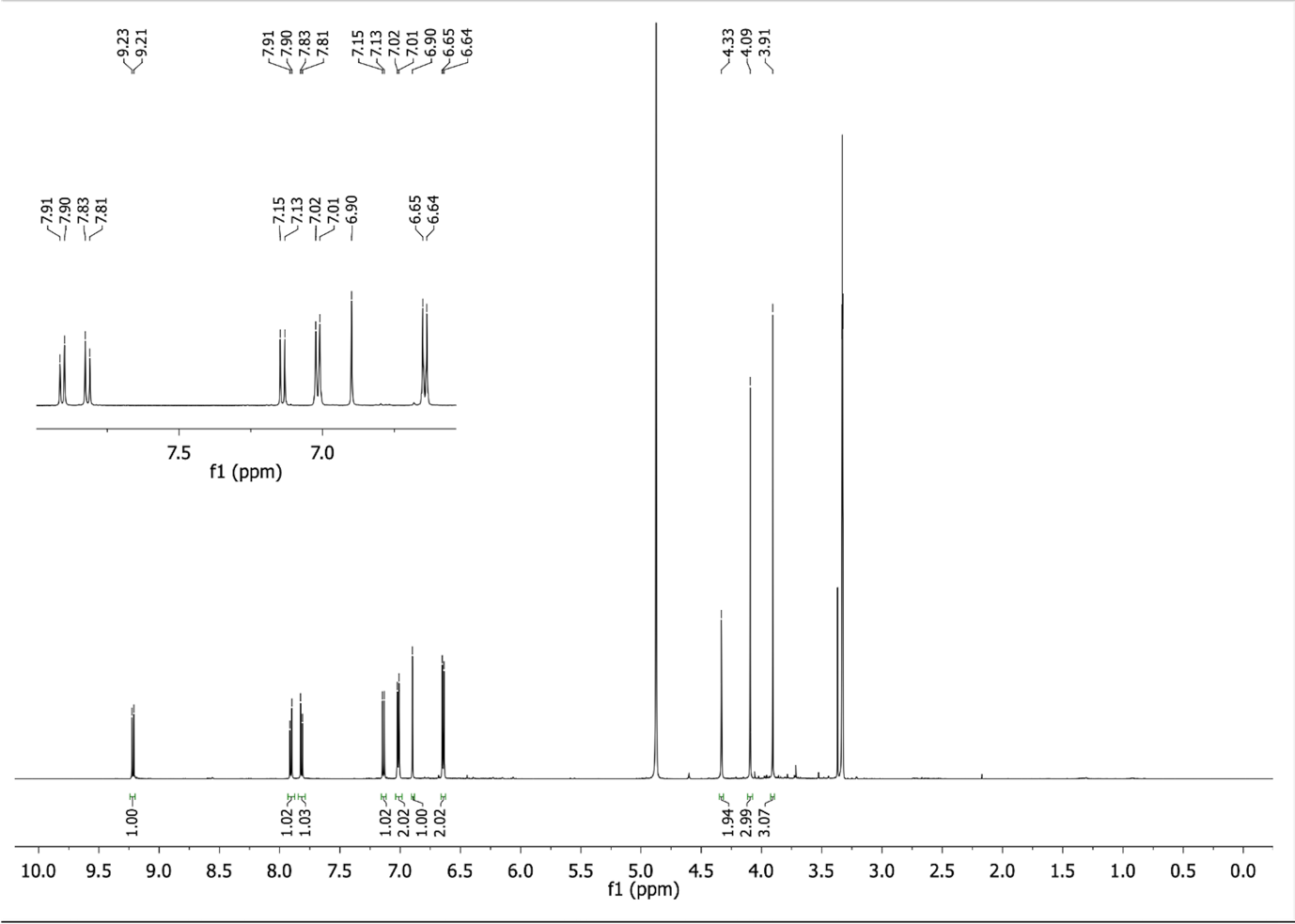
**

**2:** Table S1 Chemical structures and mass fragmentations of identifiable compounds in *E. macrobulbon* extract

**Table S1 Chemical structures and mass fragmentations of identifiable compounds in** *E. macrobulbon* **extract**

| **Cpd No.** | **Rt. (min)** | **Compound name** | **m/z** | **Chem. Formula** | **Ionized mass (m/z)** | **Proposed Structure** | **Fragmentation (Lose of m/z)** |
| --- | --- | --- | --- | --- | --- | --- | --- |
| **1** | 1.75 | Hexose | 180.0700 | C_6_H_12_O_6_ | 179.0627 [M-H]^-^ |  | 89.0285,59.0173 |
| **2** | 1.92 | Sucrose | 388.1339 | C_12_H_22_O_11_ | 387.1266 [M-HCOO]^-^ |  | 341.1172,179.0614,89.0283 |
| **3** | 3.69 | Arbutin | 286.1053 | C_13_H_18_O_7_ | 331.1142 [M-HCOO]^-^ |  | 285.0982,244.9138,161.0452,123.0447 |
| **4** | 5.88 | N-Nitroso-3-hydroxypyrolidine | 176.0750 | C_4_H_8_N_2_O_2_ | 175.0677 [M-H]^-^ |  | 115.0447,85.0697,59.0172 |
| **5** | 7.15 | 2-ethyl-6-((4,7,8-trimethoxyphenanthren-2-yl)oxy)tetrahydro-2H-pyran-3,4,5-triol | 444.1768 | C_24_H_28_O_8_ | 443.1695 [M-H]^-^ |  | 285.1054,113.0652 |
| **6** | 7.17 | 2-((6-ethyl-5-hydroxy-4-((4-hydroxybenzyl)oxy)-2-((4,7,8-trimethoxyphenanthren-2-yl)oxy)tetrahydro-2H-pyran-3-yl)oxy)-6-(hydroxymethyl)tetrahydro-2H-pyran-3,4,5-triol | 874.3348 | C_43_H_54_O_19_ | 909.3255 [M+Cl]^-^ |  | 873.3163,605.2198,443.1649,323.1064 |
| **7** | 7.32 | 4-Hydroxybenzaldehyde | 122.0418 | C_7_H_6_O_2_ | 121.0345 [M-H]^-^ |  | 92.0265,65.0396 |
| **8** | 7.69 | 2-((6-ethyl-5-hydroxy-4-((4-hydroxybenzyl)oxy)-2-((4,7,8-trimethoxyphenanthren-2-yl)oxy)tetrahydro-2H-pyran-3-yl)oxy)-6-(hydroxymethyl)tetrahydro-2H-pyran-3,4,5-triol | 712.2782 | C_37_H_44_O_14_ | 711.2709 [M-H]^-^ |  | 605.2206,443.1654,285.1054,207.0719,123.0497 |
| **9** | 8.35 | 2-((6-ethyl-4-((4-hydroxybenzyl)oxy)-5-methoxy-2-((4,7,8-trimethoxyphenanthren-2-yl)oxy)tetrahydro-2H-pyran-3-yl)oxy)-6-(hydroxymethyl)tetrahydro-2H-pyran-3,4,5-triol | 726.2952 | C_38_H_46_O_14_ | 761.2647 [M+Cl]^-^ |  | 457.1818,285.1055,153.061 |
| **10** | 8.80 | 2-ethyl-6-((2-ethyl-6-((2-ethyl-4-((4-hydroxybenzyl)oxy)-5-((3,4,5-trihydroxy-6-(hydroxymethyl)tetrahydro-2H-pyran-2-yl)oxy)-6-((4,7,8-trimethoxyphenanthren-2-yl)oxy)tetrahydro-2H-pyran-3-yl)oxy)-5-hydroxy-4-((4-hydroxybenzyl)oxy)tetrahydro-2H-pyran-3-yl)oxy)tetrahydro-2H-pyran-3,4,5-triol | 1138.4621 | C_58_H_74_O_23_ | 1173.4110 [M+Cl]^-^ |  | 1137.4331,869.3214,753.2724,485.1765,443.1654 |
| **11** | 8.91 | 2-((6-ethyl-5-((3-hydroxybenzyl)oxy)-4-((4-hydroxybenzyl)oxy)-2-((4,7,8-trimethoxyphenanthren-2-yl)oxy)tetrahydro-2H-pyran-3-yl)oxy)-6-(hydroxymethyl)tetrahydro-2H-pyran-3,4,5-triol | 818.3234 | C_44_H_50_O_15_ | 853.2932 [M+Cl]^-^ |  | 817.3453,711.2621,549.2094,443.1654,391.1459,285.1047 |
| **12** | 9.08 | 2-((6-ethyl-5-((6-ethyl-3,4-dihydroxy-5-methyltetrahydro-2H-pyran-2-yl)oxy)-4-((4-hydroxybenzyl)oxy)-2-((4,7,8-trimethoxyphenanthren-2-yl)oxy)tetrahydro-2H-pyran-3-yl)oxy)-6-(hydroxymethyl)tetrahydro-2H-pyran-3,4,5-triol | 870.3400 | C_45_H_58_O_7_ | 869.3327 [M-H]^-^ |  | 711.2623,443.1653,285.1044 |
| **13** | 9.56 | 4-methoxy-9,10-dihydro-2,7-phenanthrenediol | 242.0943 | C_15_H_14_O_3_ | 241.0881 [M-H]^-^ |  | ND |
| **14** | 10. 73 | 4,7,8-trimethoxyphenanthren-2-ol | 284.1049 | C_17_H_16_O_4_ | 283.0709 [M-H]^-^ |  | 240.0492 |
| **15** | 11.11 | 4-methoxy-2,7-phenanthrenediol | 240.0786 | C_15_H_12_O_3_ | 239.0719 [M-H]^-^ |  | ND |
| **16** | 11.46 | 8-(4-hydroxybenzyl)-1,5,7-trimethoxy-9,10-dihydrophenanthren-2-ol | 392.1387 | C_23_H_20_O_6_ | 427.1085 [M-H]^-^ |  | 281.0891,109.0336 |
| **17** | 11.53 | 1,5-dimethoxy-2,7-phenanthrenediol | 270.0892 | C_16_H_14_O_4_ | 269.0832 [M+Cl]^-^ |  | 254.0654,239.0416,211.0462 |
| **18** | 12.04 | 9,10‒dihydro‒4‒(4'‒hydroxy benzyl)‒2,5‒dimethoxyphenanthrene‒1,7‒diol | 378.1467 | C_23_H_22_O_5_ | 377.1402 [M-H]^-^ |  | 362.1162, 331.0974, 301.0865, 253.0501, 138.4688, 93.0331 |
| **19** | 12.10 | 1-(4-hydroxybenzyl)-4-methoxy-9,10-dihydrophenanthrene-2,7-diol | 348.1476 | C_22_H_20_O_4_ | 347.1399 [M-Cl]^-^ |  | 331.1054,253.0932 |
| **20** | 12.32 | 1-(4-hydroxybenzyl)-9-methoxyphenanthrene-2,7-diol | 346.1205 | C_22_H_18_O_4_ | 345.1245 [M-H]^-^ |  | 330.0977,237.062,93.0384 |
| **21** | 12.70 | **1-(4-hydroxybenzyl)-4,8-dimethoxy-2,7-phenanthrenediol (HDP)** | 376.1438 | C_23_H_20_O_5_ | 375.1361 [M-H]^-^ |  | 360.1093,317.0903,93.0383 |
| **22** | 14.25 | 4,4'-((8-hydroxy-2,4,7-trimethoxyphenanthrene-1,9-diyl)bis(methylene))dicyclohexanol | 508.1679 | C_28_H_28_O_9_ | 507.1606 [M-H]^-^ |  | 390.0984,304.9198 |
| **23** | 14.26 | 4,8,4',8'‒tetramethoxy‒(1,1'‒biphenanthrene)‒2,7,2',7'‒tetrol | 538.1795 | C_29_H_30_O_10_ | 537.1722 [M-H]^-^ |  | 507.1186,420.1078,112.9196 |
